# Supplementary material for: Pyruvate Dehydrogenase Kinase 1 inhibition mediated oxidative phosphorylation enhancement in cartilage promotes osteoarthritis progression
Source: BMC Musculoskelet Disord. 2023 Jul 20;24:597. doi: 10.1186/s12891-023-06585-6 (PMC10357736; doi:10.1186/s12891-023-06585-6)
Supplement: Supplementary file 1 — Additional file 1. [file 12891_2023_6585_MOESM1_ESM.zip › BP.Gsea.1653623667859/GOBP_REGULATION_OF_SPROUTING_ANGIOGENESIS.html]

Details for gene set GOBP\_REGULATION\_OF\_SPROUTING\_ANGIOGENESIS[GSEA]

|  || Dataset | 55457\_collapsed\_to\_symbols.cls.cls #OA\_versus\_normal.cls.cls #OA\_versus\_normal\_repos |
| Phenotype | cls.cls#OA\_versus\_normal\_repos |
| Upregulated in class | normal |
| GeneSet | GOBP\_REGULATION\_OF\_SPROUTING\_ANGIOGENESIS |
| Enrichment Score (ES) | -0.53939533 |
| Normalized Enrichment Score (NES) | -1.8802316 |
| Nominal p-value | 0.0 |
| FDR q-value | 0.059620664 |
| FWER p-Value | 0.795 |
Table: GSEA Results Summary

  

Fig 1: Enrichment plot: GOBP\_REGULATION\_OF\_SPROUTING\_ANGIOGENESIS      
 Profile of the Running ES Score & Positions of GeneSet Members on the Rank Ordered List

  

| SYMBOL | TITLE | RANK IN GENE LIST | RANK METRIC SCORE | RUNNING ES | CORE ENRICHMENT || 1 | FGF18 | fibroblast growth factor 18 [Source:HGNC Symbol;Acc:HGNC:3674] | 969 | 0.525 | -0.0323 | No |
| 2 | PIK3CB | "phosphatidylinositol-4,5-bisphosphate 3-kinase catalytic subunit beta [Source:HGNC Symbol;Acc:HGNC:8976]" | 2186 | 0.340 | -0.0990 | No |
| 3 | ALOX5 | arachidonate 5-lipoxygenase [Source:HGNC Symbol;Acc:HGNC:435] | 3776 | 0.179 | -0.2078 | No |
| 4 | PKM | pyruvate kinase M1/2 [Source:HGNC Symbol;Acc:HGNC:9021] | 3963 | 0.159 | -0.2092 | No |
| 5 | FGF1 | fibroblast growth factor 1 [Source:HGNC Symbol;Acc:HGNC:3665] | 4436 | 0.118 | -0.2362 | No |
| 6 | FGF16 | fibroblast growth factor 16 [Source:HGNC Symbol;Acc:HGNC:3672] | 5013 | 0.072 | -0.2751 | No |
| 7 | PPP1R16B | protein phosphatase 1 regulatory subunit 16B [Source:HGNC Symbol;Acc:HGNC:15850] | 6283 | -0.026 | -0.3715 | No |
| 8 | SYNJ2BP | synaptojanin 2 binding protein [Source:HGNC Symbol;Acc:HGNC:18955] | 6471 | -0.040 | -0.3828 | No |
| 9 | CEACAM1 | CEA cell adhesion molecule 1 [Source:HGNC Symbol;Acc:HGNC:1814] | 6960 | -0.077 | -0.4144 | No |
| 10 | JAK1 | Janus kinase 1 [Source:HGNC Symbol;Acc:HGNC:6190] | 6995 | -0.079 | -0.4106 | No |
| 11 | FGF2 | fibroblast growth factor 2 [Source:HGNC Symbol;Acc:HGNC:3676] | 7183 | -0.095 | -0.4173 | No |
| 12 | CREB3L1 | cAMP responsive element binding protein 3 like 1 [Source:HGNC Symbol;Acc:HGNC:18856] | 7324 | -0.104 | -0.4197 | No |
| 13 | E2F2 | E2F transcription factor 2 [Source:HGNC Symbol;Acc:HGNC:3114] | 8090 | -0.162 | -0.4658 | No |
| 14 | S100A1 | S100 calcium binding protein A1 [Source:HGNC Symbol;Acc:HGNC:10486] | 8304 | -0.178 | -0.4677 | No |
| 15 | GHSR | growth hormone secretagogue receptor [Source:HGNC Symbol;Acc:HGNC:4267] | 8472 | -0.191 | -0.4650 | No |
| 16 | TJP1 | tight junction protein 1 [Source:HGNC Symbol;Acc:HGNC:11827] | 8523 | -0.195 | -0.4529 | No |
| 17 | IL10 | interleukin 10 [Source:HGNC Symbol;Acc:HGNC:5962] | 8855 | -0.221 | -0.4605 | No |
| 18 | JCAD | junctional cadherin 5 associated [Source:HGNC Symbol;Acc:HGNC:29283] | 8989 | -0.231 | -0.4519 | No |
| 19 | SMAD1 | SMAD family member 1 [Source:HGNC Symbol;Acc:HGNC:6767] | 9905 | -0.312 | -0.4974 | No |
| 20 | EPN2 | epsin 2 [Source:HGNC Symbol;Acc:HGNC:18639] | 10446 | -0.367 | -0.5094 | Yes |
| 21 | TNN | tenascin N [Source:HGNC Symbol;Acc:HGNC:22942] | 10713 | -0.395 | -0.4977 | Yes |
| 22 | FUT1 | fucosyltransferase 1 (H blood group) [Source:HGNC Symbol;Acc:HGNC:4012] | 11086 | -0.442 | -0.4903 | Yes |
| 23 | SEMA6A | semaphorin 6A [Source:HGNC Symbol;Acc:HGNC:10738] | 11126 | -0.446 | -0.4568 | Yes |
| 24 | KLF2 | Kruppel like factor 2 [Source:HGNC Symbol;Acc:HGNC:6347] | 11350 | -0.478 | -0.4349 | Yes |
| 25 | THBS1 | thrombospondin 1 [Source:HGNC Symbol;Acc:HGNC:11785] | 11498 | -0.501 | -0.4053 | Yes |
| 26 | PDPK1 | 3-phosphoinositide dependent protein kinase 1 [Source:HGNC Symbol;Acc:HGNC:8816] | 11839 | -0.557 | -0.3861 | Yes |
| 27 | CEMIP2 | cell migration inducing hyaluronidase 2 [Source:HGNC Symbol;Acc:HGNC:11869] | 11971 | -0.588 | -0.3481 | Yes |
| 28 | ADAMTS9 | ADAM metallopeptidase with thrombospondin type 1 motif 9 [Source:HGNC Symbol;Acc:HGNC:13202] | 12428 | -0.717 | -0.3248 | Yes |
| 29 | ITGA5 | integrin subunit alpha 5 [Source:HGNC Symbol;Acc:HGNC:6141] | 12474 | -0.737 | -0.2679 | Yes |
| 30 | GLUL | glutamate-ammonia ligase [Source:HGNC Symbol;Acc:HGNC:4341] | 12527 | -0.762 | -0.2095 | Yes |
| 31 | VEGFA | vascular endothelial growth factor A [Source:HGNC Symbol;Acc:HGNC:12680] | 12629 | -0.813 | -0.1507 | Yes |
| 32 | EPN1 | epsin 1 [Source:HGNC Symbol;Acc:HGNC:21604] | 12833 | -1.037 | -0.0816 | Yes |
| 33 | KLF4 | Kruppel like factor 4 [Source:HGNC Symbol;Acc:HGNC:6348] | 12848 | -1.058 | 0.0040 | Yes |
Table: GSEA details [plain text format]

  

Fig 2: GOBP\_REGULATION\_OF\_SPROUTING\_ANGIOGENESIS      
 Blue-Pink O' Gram in the Space of the Analyzed GeneSet

  

Fig 3: GOBP\_REGULATION\_OF\_SPROUTING\_ANGIOGENESIS: Random ES distribution      
 Gene set null distribution of ES for **GOBP\_REGULATION\_OF\_SPROUTING\_ANGIOGENESIS**
